# A morphologic and semi-quantitative technique to analyze synthesis and release of specific proteins in cells

**DOI:** 10.1186/s12860-014-0045-1

**Published:** 2014-12-05

**Authors:** Guowei Huang, Yun Wang, Juping Wang, Chunzhang Yang, Tao Huang, Zhengping Zhuang, Jiang Gu

**Affiliations:** Department of Pathology and Pathophysiology, Guangdong Provincial Key Laboratory of Infectious Disease and Immunopathology, Shantou University Medical College, Shantou, Guangdong 515041 China; Surgical Neurology Branch, National Institute of Neurological Disorders and Stroke, National Institutes of health, Bethesda, Maryland 20892 USA

**Keywords:** Morphology, Protein, Amino acid, Co-localization

## Abstract

**Background:**

With the rapid advancement of cell biology, the evaluation of a given protein’s synthesis and release in cells becomes critical. However, up to now there has been no technique available to morphologically visualize and measure a newly synthesized protein in cells, nor can we measure the protein’s release from the cells.

**Results:**

In this study, we developed a set of assays combining pulse chase amino acid substitution, non-radioactive labeling, and immunofluorescence co-localization to visualize newly synthesized proteins in individual cells and then to detect their release using modified ELISA. We demonstrated the synthesis and release of Bcl-2, MMP-9, and immunoglobulin G (IgG) in a human trophoblast cell line, of which the last finding has not been reported previously.

**Conclusions:**

This new technique offers a powerful tool to evaluate the dynamics of the synthesis and release of target proteins in individual cultured cells with wide applications in genetic and protein analysis.

**Electronic supplementary material:**

The online version of this article (doi:10.1186/s12860-014-0045-1) contains supplementary material, which is available to authorized users.

## Background

There are on average about 10,000 proteins per cell with about one million copies each [1]. The dynamics of protein synthesis and release constitutes a major part of the biological activities that maintain the structure and function of a cell. Many techniques are available to detect proteins within cells, however, there has been no technique dedicated to dynamically monitor and visualize the synthesis and release of a particular protein or its peptides in individual cells. Immunohistochemistry with a wealth of specific antibodies can visualize a particular protein or peptides in its static presence in cells, but cannot yield data about the synthetic activities or follow the process of protein or peptide synthesis and release. Pulse chase assay, which incorporates radioisotope-labeled amino acid substitutes in newly synthesized proteins, can detect newly synthesized proteins in their entirety but not measure the synthesis of a particular protein/peptide. Nor can it visualize the newly synthesized proteins in cells morphologically. With increased importance of detecting genomic alternations and subsequent changes in protein synthesis, as a base for molecular and personalized medicine, and the recent discovery of a large number of gene switches, there is a surge of demand for measuring the dynamics of synthesis of a particular protein and then detecting its release in individual cells, but a reliable technique to address this need has not been available.

Recently, small bioorthogonal functional groups, especially L-homopropargylglycine (HPG) with alkyne group or L-azidohomoalanine (AHA) with azid group, a surrogate for L-methionine, have been adopted to tag proteins [2], glycans [3] and lipids [4] in cells. After giving HPG or AHA to cultured cells, the tags are incorporated into newly synthesized proteins that can be extracted from the cells. The alkyne-labeled or azide-labeled proteins can be covalently coupled to azide-biotin or alkyne-biotin that can be detected with western blot. The newly synthesized proteins can be further analyzed and identified with differential 2D electrophoresis and mass spectrometry (MS) [2,5]. This technique offers the possibility of measuring particular protein synthesis but the protocol is cumbersome, labor-intensive, and expensive, and the derived data are complicated and often confusing due to the large number of new proteins synthesized in any given cell at any given time. Although there are reports that newly synthesized total proteins can be visualized morphologically after non-radioactive labeling with a pulse chase assay [2], there is no report about the synthesis of particular proteins of interest in cells morphologically.

We developed a new technique combining non-radioisotope labeling of amino acid substitute, pulse chase assay, azide-Alex555 or azide-biotin labeling reaction, immunofluorescence co-localization, and image analysis that can visualize the synthesis of any particular protein morphologically in cultured cells, and then detect its release semi-quantitatively with ELISA. We used a human placental trophoblast cell line (TEV-1) as a model and demonstrated the synthesis and release of three proteins Bcl-2, MMP-9, and IgG, of which the last finding has not been reported previously. The reliability and reproducibility of this technique were verified with extensive controls including down-regulation of the above three target proteins with inhibition of their mRNA expressions with siRNA post-transfection. By varying the timing and duration of labeled amino acid incorporation, the dynamic changes of a particular protein synthesis and release can be traced to evaluate their activities.

## Results

The results of the technique provide images of a number of activities within the positive cells of interest. The newly synthesized protein of interest was demonstrated with a distinct color labeling resulting from the overlapping of two colors, one delineating total proteins of interest and another displaying total newly synthesized proteins. The co-localization of the two color images gives rise to a third color demonstrating the amount and location of the newly synthesized protein of interest. The total portion of the particular protein of interest was provided by the results of immunoflourence staining with specific antibodies. The positive staining would represent both previously synthesized and newly synthesized portion of this protein. The total newly synthesized protein was demonstrated by the Alex555 labeling derived from the newly incorporated amino acid substitute during the synthesis of new proteins in the cells. The positive signals of this reaction would represent the total proteins of all kinds. The overlapping portion as demonstrated by the collaboration of the two distinct fluorescent colors, green for total existence of the protein of interest and red for the total newly synthesized protein, gave rise to a third color, in this case white, that demonstrated the newly synthesized protein of interest. The overlapping portion can be calculated by measuring the portions of the two fluorescent color co-localization of the morphological image with an image analyzing software. The controls of the technique were provided by the routine controls for immunofluorescence staining and pulse chase assay and a siRNA inhibition technique to specifically decrease the synthesis of the particular protein of interest, as described in the following section. The release of the newly synthesized proteins was measured with modified ELISA by detecting their concentration in the supernatant. The dynamics of their release was demonstrated.

### General description of the technique

The technique consists of seven steps. 1. Administration of labeled amino acid substitute, HPG or AHA, into cultured cells seeded on slides with culture medium deprived of the replaced amino acid, in this case L-methionine. 2. After a pre-determined duration, label the incorporated amino acid substitute with Alex555 (red florescence) for immunofluorescence of newly synthesized proteins in cells or with biotin for the detection of their release into the supernatant. 3. Detect the Alex555 labeling with immunoflorescence to visualize the total newly synthesized proteins on the glass slide with a confocal microscope. 4. Detect the particular proteins of interest with specific antibodies and immunoflorescent staining with a different color (Alex448, green florescence). 5. Generate double labeling image with a confocal microscope and identify co-localization of the particular proteins of interest and the total newly synthesized proteins with an image analyzing software, and then calculate the percentage of the newly synthesized target proteins in individual cells. 6. Varify the specificity of immunofluorescence co-localization with siRNA interference assay combined with morphological visualization to show that the newly synthesized target proteins were declined in quantity when their mRNAs were inhibited by the specially designed RNAi for the particular proteins. 7. Detect the newly synthesized target proteins in the supernatant captured by primary antibody coated on the wells followed by biotin label with a modified ELISA analysis and describe the dynamics of their release.

### Newly synthesized proteins, including total proteins, Bcl-2, MMP-9, and IgG, detected in cultured cells with western blot

**Figure 1:**
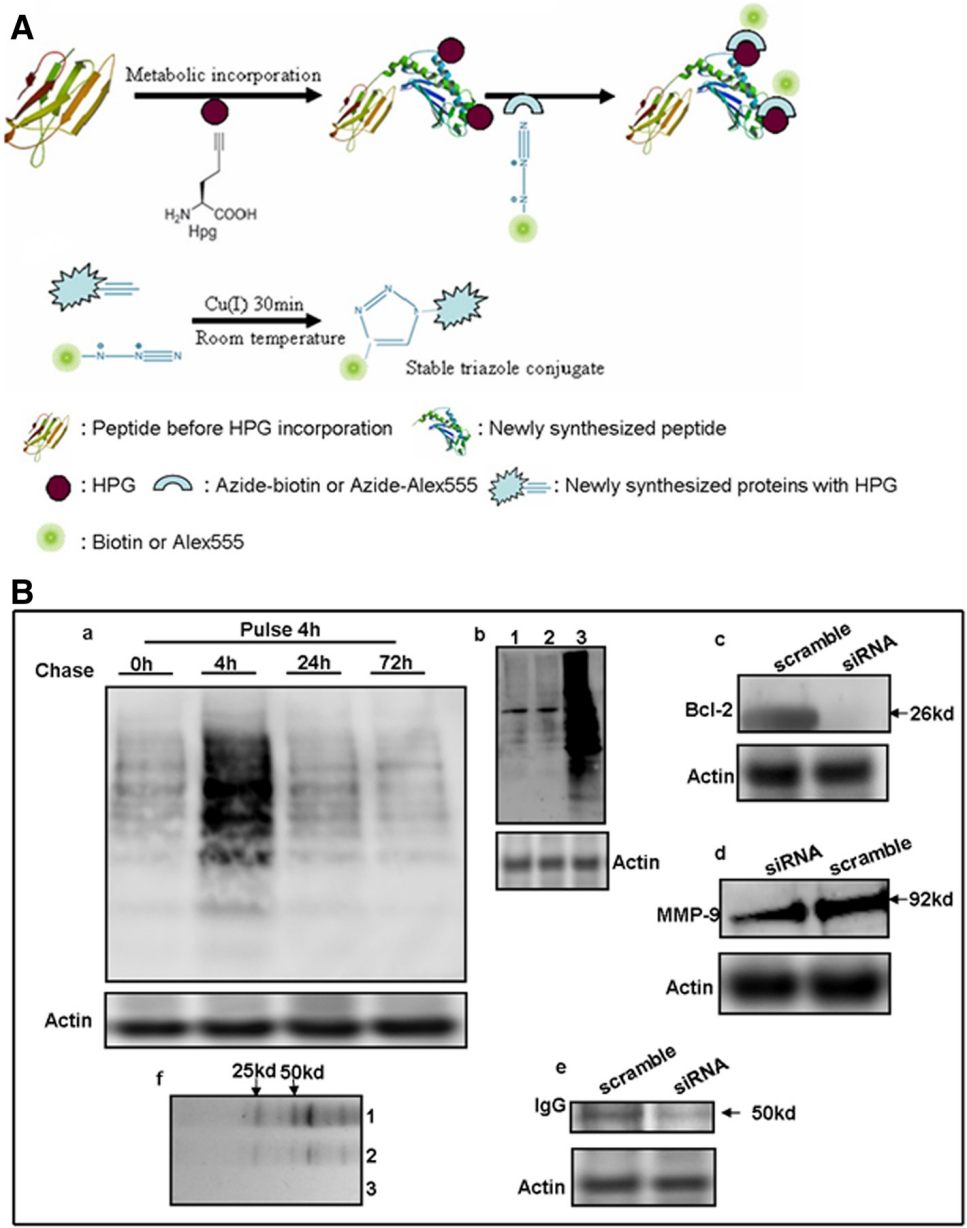
**Schematic diagram showed non-radioactive metabolic incorporation followed by azide-biotin or azide-Alex555 labeling, and biotin signals of proteins were detected by streptavidin-HRP by western blot.** HPG is incorporated into newly synthesized proteins by metabolism and protein synthesis and the triazole conjugation between newly alkyne proteins labeled HPG and azide labeled either biotin or Alex555 via CuSO4 catalysis **(A)**. **(B-a)** The detection of biotin signals from extracted total proteins labeled by labeling reaction. Normal culture medium was changed to replace DMEM free of L-methionine supplemented with HPG after pulse 4 hr, and proteins were extracted in each of group at various time points including 0, 4, 24 and 72 hr. **(B-b)** Biotin signals of total proteins were detected. 1: Normal culture condition group; 2: HPG plus anisomycin group; 3: HPG group. **(B-c,d,e)** Biotin signals of Bcl-2, MMP-9 and IgG were individually detected in the immunoprecipitate pulled down by primary antibodies via siRNA post-transfection followed by non-radioactive metabolic labeling. **(B-f)** Radioactive isotope ^35^S-methonine incorporated into synthesized IgG purified by immunoprecipitation was detected by autoradiography. 1: ^35^S-methonine treated human choriocarcinoma cell line BeWo group and then antibody against human IgG immunoprecipitated human IgG in extracted total proteins; 2: cycloheximide plus ^35^S-methonine treated BeWo group then antibody against human IgG immunoprecipitated human IgG in extracted total proteins; 3: ^35^S-methonine treated skin fibroblast and then antibody against human IgG immunoprecipitated human IgG in extracted total proteins.

To establish the technique, we first determined the synthesis of total proteins, Bcl-2, MMP-9, and IgG in cell extracts with non-radioactive labeling of pulse-chase assay. The principle of HPG metabolic incorporation and labeling reaction is shown in the Figure 1A [6]. Briefly, HPG incubation in DMEM free of L-methionine lasted for 4 hr and chased for 4 hr in normal culture medium. Total protein was extracted and labeled with azide-biotin in a CuSO_4_ catalystic reaction between azide from azide-biotin and alkyne from HPG. Biotin signals were detected with streptavidin-HRP with western blot. If the biotin signals from newly synthesized proteins were detected, the evidence of new protein synthesis in cells was obtained. The results of our experiment showed that HPG was successfully incorporated into newly synthesized proteins and Bcl-2, MMP-9, and IgG were synthesized in TEV-1 cells (Additional file 1, Figure 1B-a,-b,-c,-d,-f). Especially, newly synthesized IgG was further proved by radioactive pulse chase (Additional file 1, Figure 1B-e) and IgG mRNA transcription was also further demonstrated by RT-PCR (Additional file 1: Figure S1).

### Bcl-2 and IgG mRNA levels in cultured cells detected with real time RT-PCR post-siRNA transfection

**Figure 2:**
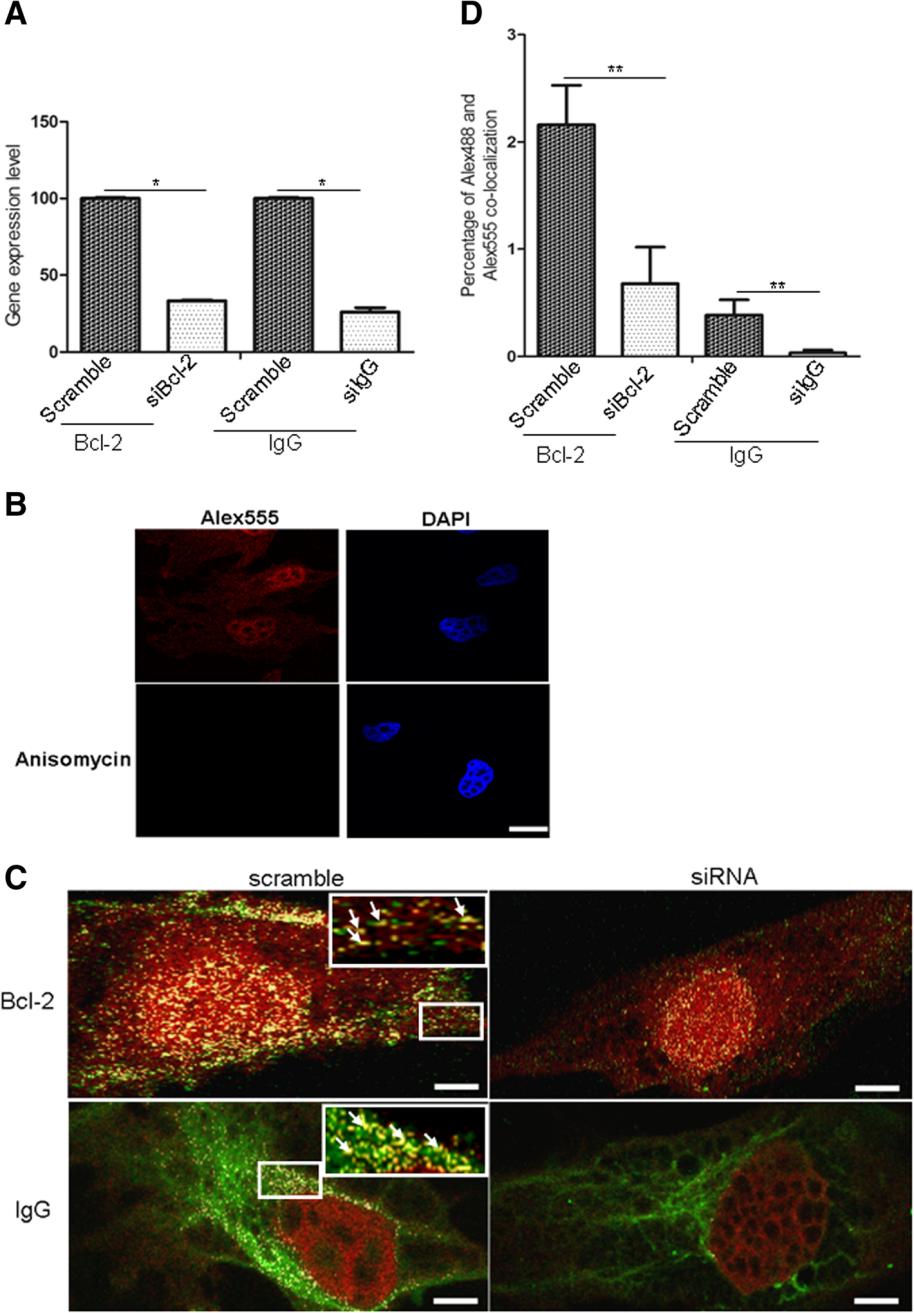
**The transcripts of target gene Bcl-2 and IgG post-siRNA transfection were detected and immunofuorescent co-localization analysis of Alex488 and Alex555 was performed. (A)** The transcripts of target gene Bcl-2 and IgG post-siRNA transfection 48 hr were detected in TEV-1 cells by real time RT-PCR. **(B)** Immunofluorescence signals for Alex555 (red) were observed with a Zeiss laser confocal microscope at 546 nm emition wavelength in HPG and HPG plus anisomycin treated group. Nuclear staining was performed with DAPI. **(C)** Immunofuorescent co-localization analysis of Alex488 (green) and Alex555 (red). White dots, as arrows were shown in big magnification, represented newly synthesized Bcl-2 and IgG proteins, respectively. **(D)** The percentage of immunofluorescence co-localization of Alex488 and Alex555 in TEV-1 cells was detected. Bar: 5 μm; Magnification: 10 ×100. Data are presented as means ± S.E. of three independent experiments (*P < 0.05; **p < 0.01).

To acquire effective siRNA oligos to be used for testing the specificity of immunofluorescence co-localization, mRNA levels of the two genes were measured with real time RT-PCR (Figure 2A). The results showed that effective siRNA oligos were acquired. The details were described in Additional file 1.

### Immunofluorescence staining to detect total protein synthesis *in situ* morphologically

HPG was incorporated into newly synthesized proteins and subsequently labeled with azide-Alex555. Immunofluorescence signals were observed with a Zeiss laser confocal microscope at 546 nm emittion wavelength. The results showed the total newly synthesized proteins in the cytoplasma and the nucleus (Figure 2B, Alex555 group). To establish the specificity of immunofluorescence staining, a HPG protein synthesis inhibitor, anisomycin, was added to culture medium and the immunoinfluorescnt signals were completely abolished (Figure 2B, Anisomycin group).

### Immunofluorescen co-localization analysis to detect target protein synthesis *in situ* morphologically

On the same preparation of cytospin slides used for detecting total newly synthesized proteins described above, we respectively detected the two target proteins (Bcl-2 and IgG) with immunofluorescence (Alex488, green florescence) with primary antibodies and secondary antibody (goat anti-mouse IgG-Alex488) and IgG was directly detected with mouse anti-human IgG-Alex488 diluted with labeling reaction buffer. Immunoreactivities of the two proteins were readily demonstrated in the cytoplasm or nuclear of the cell line. On the slide preparation, three overlapping photographs were obtained, i.e. red florescence to demonstrate the total newly synthesized protein, green florescence to demonstrate the existence of the particular protein of interest, and the white florescence derived from the collaboration of the red and the green color to display the location and quantity of the newly synthesized protein of interest (Figure 2C). After siRNA transfection, the positive fluorescent signals were markedly declined in comparison to the scramble control (Figure 2C). We calculated the percentage of white pixels, representing Alex488 and Alex555 co-localization, against the total pixel area of the cytoplasm of each cell. The percentage of white pixels of the siRNA treated cells was compared to that of the scrambled control group where no inhibition of protein synthesis was induced. The percentage of immunofluorescence co-localization of Alex555 and Alex488 for Bcl-2 was reduced by 3.2-fold in the siRNA group in comparison to the Bcl-2 scramble group, and the percentage of immunofluorescence co-localization of IgG was reduced by 11.7-fold in comparison to the control group (Figure 2D; Table 1).

**Table 1:** **The results of statistical analysis for immunofluorescence co-localization**

	**Scramble siRNA**	**Bcl-2 siRNA**	**Scramble siRNA**	**IgG siRNA**
AVE	2.161	0.677	0.386	0.033
SE	0.366	0.341	0.143	0.027
P	0.002		0.04	

AVE: Average of the percentage of immunofluorescence co-localization; SE: standard errors.

### Newly synthesized target proteins released from cultured cells

**Figure 3:**
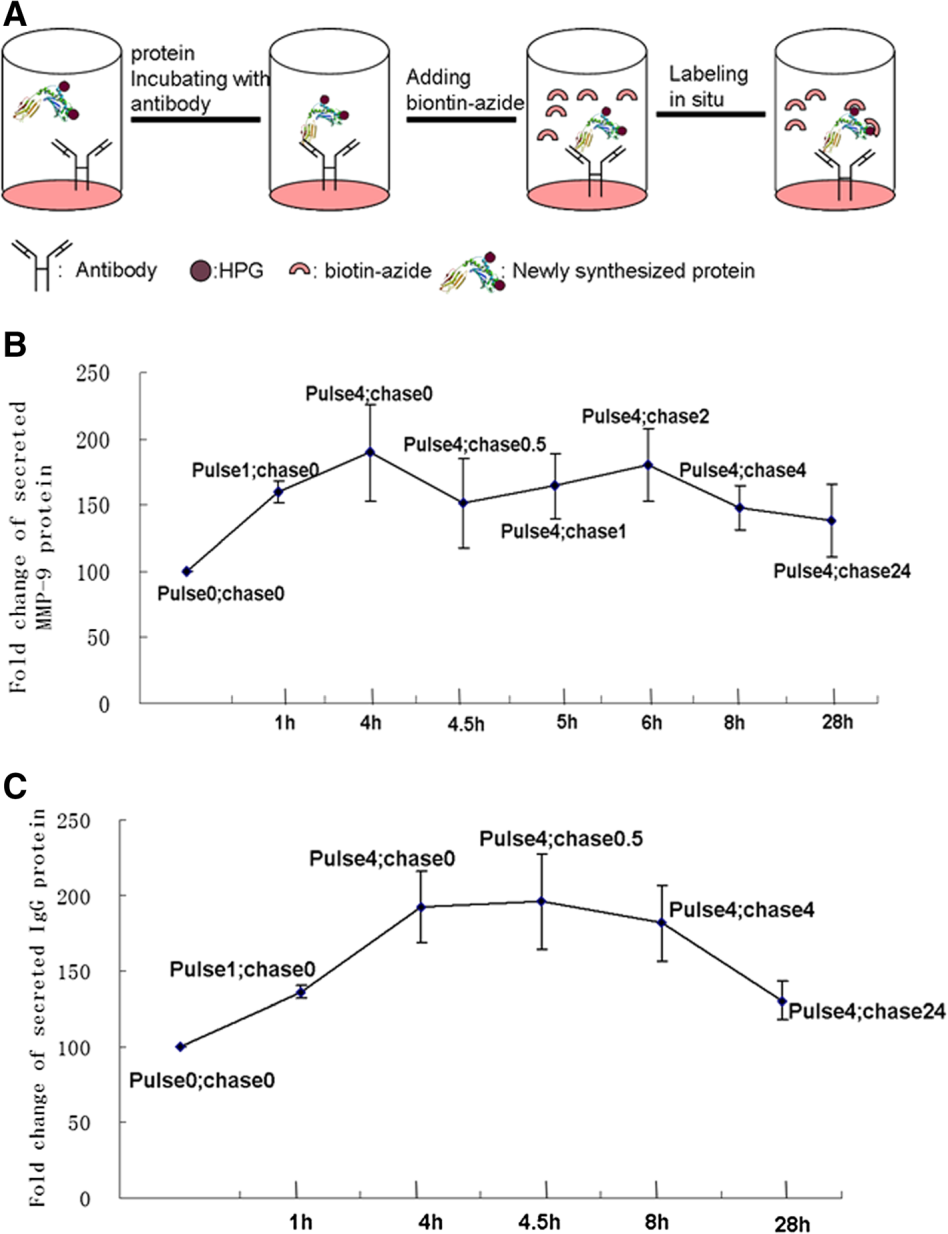
**Newly synthesized protein secretion was detected. (A)** The schematic diagram illustrated the detection of newly synthesized protein release in the culture supernatant. Newly synthesized proteins from HPG group were labeled with azide-biotin *in situ,* by clicking its reaction, the biotin signals were incubated with streptavidine and measured by an ELISA reader. **(B)** The dynamic curve for newly synthesized MMP-9 proteins was set up corresponding to various time points by adapted ELISA method. Normal culture condition was treated as negative control and selected as baseline and named as pulse 0; chase 0. Pulse 1 h, chase 0 h named as pulse 1; chase 0. Pulse 4 h, chase 0 h named as pulse 4; chase 0. Pulse 4 h, chase 0.5 h named as pulse 4; chase 0.5. Pulse 4 h, chase 2 h named as pulse 4; chase 2. Pulse 4 h, chase 4 h named as pulse 4; chase 4. Pulse 4 h, chase 24 h named as pulse 4; chase 24. **(C)** The dynamics curve for newly synthesized IgG protein was set up corresponding to various time points. Normal culture condition was treated as negative control and selected as baseline, and the named method for various time points was similar to MMP-9.

Combined with diluted IgG-biotin conjugate, we measured serial OD values in response to serially diluted IgG-biotin conjugate with Streptavidine-HRP with a modified ELISA method. The IgG-biotin conjugate was diluted to 224 ng/ml, 112 ng/ml, 56 ng/ml, 28 ng/ml, 14 ng/ml, 0 ng/ml (Table 2). We detected the release of IgG in TEV-1 cell culture supernatant and demonstrated that there was newly synthesized IgG in culture supernatant by a modified ELISA (Figure 3A). Newly synthesized IgG release was prevented by addition of HPG plus anisomycin. The OD value of negative controls, which included normal culture medium and PBS, was the same to the background OD value (Table 3). Alternatively, we measured IgG secretion in TEV-1 cell culture supernatant with a commercial human IgG ELISA kit (Immunology Consultants Laboratory, Oregon, USA) and IgG release in supernatant was undetectable. Therefore, our method of detection for newly synthesized target protein release was more sensitive than the commercially available ELISA kit.

**Table 2:** **OD value of serial diluted IgG-biotin standard detected by ELISA**

OD value	0.083	0.1215	0.2569	0.7856	2.1300	3.8900
Concentration (ng/ml)	0	14	28	56	112	224

Serial diluted IgG-biotin conjugate was incubated with streptavidine-HRP and OD value was detected after 3, 3′, 5, 5′tetramethylbenzidine color development and 2 M H_4_SO_4_ termination reaction. n = triplicate.

**Table 3:** **OD value of secreted IgG detected by ELISA in cell culture supernatant**

	**Normal culture condition**	**HPG**	**HPG plus anisomycin**	**Normal culture medium**	**PBS**
OD value	0.078	0.1473	0.0799	0.0800	0.0820

Newly synthesized IgG in culture supernatant was detected by non-radioactive metabolic method combined with an adapted ELISA method. Triplicate for each sample. n = triplicate.

Likewise, newly synthesized MMP-9 protein with HPG was also secreted into culture supernatant and captured with mouse anti human MMP-9 coated on the wells and labeled with azide-biotin. The biotin labeled MMP-9 was incubated with streptavidine-HRP and biotin signals of MMP-9 were detected with an adapted ELISA. Combined with serially diluted MMP-9 labeled with biotin, the results showed that the OD value of MMP-9 protein from supernatant of HPG group was over the background level and the OD value of supernatant from HPG plus inhibitor, culture medium and PBS group was lower than the background OD value, indicating that TEV-1 cell line secreted newly synthesized MMP-9 protein (Table 4). The release of newly synthesized MMP-9 was also preventable by adding anisomycin (Table 5).

**Table 4:** **OD value of serial diluted MMP-9-biotin standard detected by ELISA**

OD value	0.0800	0.2700	0.4700	0.7400	1.3600	2.1900
Concentration (ng/ml)	0	10	20	40	80	160

Serial diluted MMP-9-biotin conjugate was incubated with streptavidine-HRP and OD value was detected after 3, 3′, 5, 5′tetramethylbenzidine color development and 2 M H_4_SO_4_ termination reaction. n = triplicate.

**Table 5:** **OD value of secreted MMP-9 detected by ELISA in cell culture supernatant**

	**Normal culture condition**	**HPG**	**HPG plus anisomycin**	**Normal culture medium**	**PBS**
OD value	0.2977	0.6009	0.2429	0.2992	0.0849

Newly synthesized MMP-9 in culture supernatant was detected by non-radioactive metabolic method combined with an adapted ELISA method. Triplicate for each sample. n = triplicate.

To detect the dynamics of newly synthesized protein release, we calculated the changes in OD values of HPG treated and untreated samples according to the equation for “fold change = A/N” (“A” is OD value of the testing sample and “N” is OD value of the untreated negative control). The change of dynamics was illustrated with a curve with X-axis representing various time points and Y-asix representing changes in quantity. On evaluating the dynamics of newly synthesized MMP-9 release, we found that it was increased from 1 h to 4 h following adding HPG to DMEM free of L-methionine and increased from 0.5 h to 2 h following adding normal culture media. In the HPG group, its release was gradually declined from 4 h to 24 h (Figure 3B). We also detected the dynamics of IgG release which was similar to that of MMP-9 (Figure 3C).

### Statistical analysis

Statistical analysis was described in Additional file 1.

## Discussion

To examine the expression profile of target proteins of interest in specific cell type was very important to provide the direct evidence for target gene expression from mRNA translation to protein synthesis in a given cell type. Previously, newly synthesized proteins can only be visualized in their totality with confocal microscope [7], and visualization of target proteins of interest in cells has not been possible. Now, we have established a non-radioactive method to evaluate target protein synthesis in intact cells morphologically and measured their release semi-quantitatively.

Immunohistochemistry and *in situ* hybridization have been used extensively to demonstrate the occurrence and distribution of a particular protein or peptide antigen morphologically. These techniques, however, cannot distinguish existing molecules from newly synthesized molecules. In addition, certain proteins and peptides can be phagocytized into cells or internalized via specific receptors, such as transpassing of IgG into placental trophoblasts, it is not possible to distinguish locally synthesized proteins from proteins with an extracellular origin. IgG in trophoblasts would be a good example [8]. Also, the study demonstrated that IgG was expressed in TEV-1 cells by immunochemistry, *in situ* hybridization and immunofluorescence [9]. With the new technique, we were able to establish that IgG can be synthesized in placental trophoblasts in addition to being transported from the maternal blood pool. Details of this discovery will be published separately.

The specificity of this combined technique has been established with a number of controls. In addition to the usual controls for immunostaining, western blotting, and ELISA, we used siRNA to specifically inhibit the expression of a target protein of interest. The results showed unequivocally that the reactions were specific and reproducible. Therefore, this technique, for the first time, provides a tool to evaluate the synthesis and release of a particular protein in intact cells morphologically and semi-quantitatively.

## Conclusions

The ability to morphologically demonstrate the synthesis of a particular protein in intact cells and then measure its release provides a powerful tool to investigate many biological and cellular events that cannot be evaluated effectively otherwise. The potential applications of this technique can be summarized into the following aspects.

First, it can determine the synthetic activity of a given protein in a particular cell type. Apparently the demonstration of the presence of the particular protein of interest in the cells with immunohistochemistry or immunofluorescence is not a sufficient evidence for recent protein synthesis as the proteins can be internalized from outside of the cells or may have stayed in the cells for a long time. Currently this can be demonstrated only by *in situ* hybridization to show the presence of relevant mRNA in the cells of interest. Northern blot and RT-PCR can also detect the mRNA but will not correlate to tissue and cell morphology. The *in situ* hybridization data are also not ideal as they offer no clue as to how long the mRNA has been presented in the cells and if it is newly transcribed or left over from old synthetic activities. Pulse chase assay combined with western blot or ELISA offers definitive proof of local synthesis but does not generate data concerning the cell types that actually synthesize the protein. In this regard, the new technique will provide the answer to the critical question of which cell synthesizes which protein and at what rate. The detection of IgG production by placental trophoblasts is a good example of this technical strength. The trophoblast cells are known to uptake IgG from the adjunct blood pool of the mother with specific Fc receptors and transport them to the fetus side. The new technique would be able to demonstrate that the trophoblasts are indeed able to synthesize IgG themselves in addition to internalizing IgG from the adjacent maternal blood pool. With this new technique, the two portions of IgG, i.e. the locally produced and externally transported, in the same trophoblast cells can be visualized and distinguished from each other.

This technique also enables investigators to trace the protein synthesis events chronically so as to demonstrate the time needed for a particular protein synthesis to begin, to reach its peak, and to decline. It can also detect if a particular protein is released from a particular cell type and at what rate in order to assess its possible functions. It can also trace this newly synthesized and tagged protein to assess its reactivity with other molecules such as receptors, antigens, antibodies, enzymes or other factors.

By manipulating gene expression with siRNA, up or down regulation, the corresponding proteins would change their expression profile. The new technique would be able to provide accurate information about the protein synthesis and release dynamically and semi-quantitatively. The morphological demonstration of a newly synthesized protein and its release can be examined together or separately as needed.

As the evaluation of the expression profile of individual proteins becomes more important in developing personalized medical diagnosis and treatment, the current technique would find more applications in clinical service and related research.

## Methods

### Non-radioactive metabolic labeling

Human first trimester extravillous trophoblast cell line TEV-1 (ATCC, maryland, USA) were cultured in DMEM/F12(Hyclone, Logan, USA) medium. Metabolic labeling experiment for cells was performed until 80-90% confluence as described [2]. After incubating cells with DMEM free of L-methionine (Invitrogen, California, USA) for 30 min at 37°C, 5% CO_2,_ HPG (Invitrogen, California, USA) at final concentration 50 μM in culture medium, was added to DMEM free of L-methionine for 4 hr. The above DMEM with HPG was changed into normal medium for 4 hr. Normal culture condition was served as negative control. HPG plus 40 μM anisomycin (Sigma, Missouri, USA), a protein synthesis inhibitor, were added to cultured cells as control. HPG was incubated with cultured cells for 4 hr and then changed into normal culture medium for 0, 4, 24, and 72 hr. Cell proteins were extracted and labeled by biotin and the newly synthesized Bcl-2, MMP-9, and IgG were detected by biotin signal detection and the detailed procedure was described in Additional file 1.

### RNAi for Bcl-2, MMP-9, and IgG and the detection of the three gene mRNA level with real time RT-PCR and newly synthesized Bcl-2, MMP-9, and IgG proteins detected by immunoprecipitation

siRNA oligos were designed according to IgG VDJ mRNA sequence(AY270190.1) in cancer cells on NCBI database (http://www.ncbi.nlm.nih.gov/) submitted by Qiu [10] and the sequence of siRNA oligos for Bcl-2 and MMP-9 were from references [11,12]. All the siRNA oligos were synthesized by GenePharm Company (Shanghai, China).

Cell transfection was performed as the method by Brian Dalbyb [13]. Each sample was collected post-transfection and extracted total RNA concentration was detected with Nanodrop-2000c (Thermo, Massachusetts, USA) and the procedures of real time RT-PCR were performed. The sequence of siRNA oligos and the primers for real time RT-PCR were shown in the following Table 6. The concentration of extracted proteins was detected with BCA kit and labeled by azide-biotin and immunoprecipitation for Bcl-2, MMP-9 and IgG was performed as the method above.

**Table 6:** **siRNA oligos and the primers for real time RT-PCR**

**Gene**	**siRNA(5′-3′)**	**Primer pairs(5′-3′)**	**Size(bp)**
Bcl-2	GUACAUCCAUUAUAAGCUGdtdt	GTACCTGAAACCGGCACCTGCAC	150 bp
	CAGCUUAUAAUGGAUGUACdtdt	GCTCCACAGCCTCCCATTGCC	
MMP-9	CAUCACCUAUUGGAUCCAAdtdt	CAGCCGGGACGCAGACATCG	128 bp
	UUGGAUCCAAUAGGUGAUGdtdt	GGGCGTCTCCCTGAATGCCG	
IgG	CCAAGGACACCCUCAUGAUdtdt	GTGACGGTGTCGTGGAACT	94 bp
	AUCAUGAGGGUGUCCUUGGdtdt	ACGCTGCTGAGGGAGTAGAG	
Scramble	UUCUCCGAACGUGUCACGUdtdt		
	ACGUGACACGUUCGGAGAAdtdt		
GAPDH		TAGAGCGGCCGCCATGTTGC	123 bp
		CCTGACTTCCCCGCCACACGC	

### Immunofluorescence double staining for Alex488 and Alex555

TEV-1 cells were grown on cover slides and non-radioactive metabolic labeling was performed. Immunofluorescence staining followed by labeling reaction by azide-Alex555 (Invitrogen, California, USA) was performed. Cells were fixed with 4% Paraformaldehyde in PBS and perforated by 0.25% Triton X-100 in PBS and block with normal mouse serum at room temperature for 1 hr and incubated with mouse anti human Bcl-2 at 4°C overnight and Alex488-goat anti mouse IgG as secondary antibody (Cell Signal Technology, Boston, USA). Alex488-mouse anti human IgG (Invitrogen, California, USA) was directly used to detect IgG protein in cells. *In situ* labeling from azdie-Alex555 for each sample was performed according to kit procedure (Invitrogen, California, USA). Immunofluorescence staining was observed and taken photos by Zeiss laser confocal microscope with 100× oil lens at 405, 488, and 546 nm emition wavelength.

### Immunofluorescence co-localization analysis by Imaris software and the detection of the specificity for immunofluorescence co-localizaiton by RNA interference

Immunofluorescence double staining for Alex488 for Bcl-2 and IgG and Alex555 for newly synthesized proteins was performed as the method above siRNA post-transfection. Serial planes as 1 μm thickness were acquired by Zeiss confocal microscope and the percentage of immunofluorescence co-localization of the very median plane was calculated by Imaris 6.3.1 software (Bitplane, Zurich, Switzerland). The threshold of two of labels was set to 30% of the highest intensity value for Bcl-2 and IgG to distinguish them from non-specific staining [14]. RNA interference technology was used to detect the specificity for immunofluorescence co-localization.

### The detection of the release of newly synthesized target proteins with non-radioactive metabolic labeling combined with modified ELISA

TEV-1 cells were grown on the twenty-four well plate, HPG at final concentration 50 μM was added to DMEM free of L-methionine and incubated for 4 hr and changed into 500 μl normal culture medium overnight. HPG plus anisomycin, normal culture condition, normal culture medium, and PBS were set up as negative control for ELISA. 500 μl culture supernatant was concentrated and adjusted into 100 μl volume and MMP-9 was incubated with mouse anti human coated on the enzyme strips at room temperature for 1 hr and labeled *in situ* with azide-biotin. *In situ* labeling from azdie-biotin for each sample was modified according to kit procedure (Invitrogen, California, USA). Briefly, 500 μl volume per labeling reaction was composed of 440 μl reaction buffer, 10 μl CuSO_4_, 50 μl reaction additive and azide-biotin at final concentration 5 μM and labeling reaction and final 100 μl labeling reaction solution was added to each well and incubated at room temperature for 30 min. The biotin signals of MMP-9 were detected with streptavidin-HRP (Thermo, Massachusetts, USA) and OD value for each sample was detected by modified ELISA. Commercial MMP-9 standard or IgG standard (Biosythesis biotechnology, Beijing, China) was labeled with biotin according to the procedure of AnaTag™ biotin microscale protein labeling kit (AnaSpec, California, USA). Briefly, 1 mg standard was mixed with 1/10 volume reaction buffer and incubated with biotin-X SE in DMSO at room temperature for 30 min and the biotin-protein conjugates diluted in PBS was purified with desalting column and the eluent was concentrated and adjusted to 100 μl volume and the concentration of standards was detected and diluted into several serial concentrations. Each diluted standard was incubated with primary antibody coated on the well and streptavidin-HRP and OD value for each sample was detected by ELISA. To explore the dynamics of the release of newly synthesized proteins, supernatant in cultured cells was collected at various time points from 0-24 hr after HPG incorporated into proteins, and OD value for newly synthesized target proteins in collected supernatant was detected by modified ELISA above.

### Statistical analysis

Statistical analysis was performed for the results of real time RT-PCR for target gene message RNA level pre-transfection and post-transfection and the percentage of immunofluorescence co-localization of Alex488 and Alex555 using t-test by Excel software with significance set at 0.05. In analysis where siRNA and scramble groups were compared, analysis of variance was performed.

## Additional file

Additional file 1:
**Supplement methods and results.**

## Abbreviations

IgG: Immunoglobulin G
HPG: L-homopropargylglycine
AHA: L-azidohomoalanine
