# Supplementary material for: A morphologic and semi-quantitative technique to analyze synthesis and release of specific proteins in cells
Source: BMC Cell Biol. 2014 Dec 5;15:45. doi: 10.1186/s12860-014-0045-1 (PMC4261763; doi:10.1186/s12860-014-0045-1)
Supplement: Additional file 1: — Supplement methods and results. [file 12860_2014_45_MOESM1_ESM.doc]

**A morphologic and semi-quantitative technique to analyze synthesis and release of specific proteins in cultured cells**

Guowei Huang, Yun Wang, Juping Wang, Chunzhang Yang, Tao Huang, Zhengping Zhuang, Jiang Gu

**Supplement** **methods and results**

**Methods**

**Non-radioactive labeling**

Cell proteins were extracted with T-PER lysis buffer (Thermo, Ohio, USA) and detected by BCA kit (Thermo, Massachusetts, USA) and performed in vitro labeling according to labeling kit instructions (Invitrogen, California, USA). Protein recovery was performed as the procedure Invitrogen Company provided online. 600 μl methonal and 150μl chloroform and 400 μl 18 MΏ milliQ water were added to reaction mixture above. Vortex briefly and centrifuge at 15000 xg for 5 min at 4°C and wash by methonal and carefully remove upper aqueous phase after centrifuge. Protein pellets were dried in air for 15 min and resuspended in T-PER lysis containing protease inhibitor (Roche, Basel, Switzerland).

**The detection of biotin signals from extracted total protein and Immunoprecipitation for Bcl-2, MMP-9, and IgG from proteins labeled by biotin-azide**

Labeled total proteins in loading buffer at 70°C for 10 min were directly load and run on 10% SDS-PAGE gel. Electrophoresis was carried out at 120 voltages and electroblotted at 90 voltages for 90 min. PVDF membranes were blocked for 1 hr with superblock solution (Thermo, Ohio, USA) containing 0.25% Tween-20. Biotin signals of total proteins on membrane were detected with streptavidin-HRP (Thermo, Ohio, USA) diluted as 1:100000 with Superblock solution containing 0.25% Tween-20 and exposure to Super-signal west femto (Thermo, Ohio, USA) with Alpha Innotech gel imaging systems (Cell Biosciences, California, USA)[1]. Total proteins extracted from cultured cells at HPG incubated for 4 hr and normal culture medium for 4 hr were used to immunoprecipitate Bcl-2, MMP-9, and IgG. Labeled total proteins resuspended in 200 μl T-PER containing protease inhibitor were respectively incubated with mouse anti-human Bcl-2, mouse anti-human MMP-9 (Santa Cruz, California, USA) at 4°C overnight and precipitated by magnetic beads coupled protein G (Cell Signal Technology, Boston, USA). For IgG immunoprecipitation, magnetic beads coupled protein G were directly added to in vitro labeled total proteins. HPG plus protein synthesis inhibitor anisomycin and normal culture condition were set up as negative control. After extensive washing with 0.01M PBS, all the samples were directly resolved in loading buffer and denatured at 70°C for 10 min. Biotin signals from the samples were detected with western blot above. Actin was used as a loading control and western blot was performed as the following condition: mouse anti human actin (Santa Cruz, California, USA) incubation at 4°C overnight after 5% non-fat milk (Sangon, Shanghai, China) block and exposure to ECL (Pierce, Illinois, USA) with Alpha Innotech gel imaging systems.

**Metabolic ^35^S-methionine labeling for IgG synthesis**

Metabolic ^35^S-methionine labeling was performed as previously described [2]. BeWo human choriocarcinoma cells and skin fibroblast cells (ATCC, Maryland, USA) were starved with methionine depleted medium followed by labeled in methionine-free DMEM supplemented with ^35^S-methionine for 4 hr. As control, Cycloheximide at final concentration 10 μg/ml was added to methionine-free DMEM supplemented with ^35^S-methionine. Cultured skin fibroblasts were chosen as negative control and also labeled by ^35^S-methionine. All total proteins in each of group were extracted with RIPA lysis buffer (Thermo, Massachusetts, USA) and precipitated using Dynabeads Protein G immunoprecipitation kit (Invitrogen, California, USA). Antibodies against human IgG and mouse IgG (Jackson ImmunoResearch laboratories, Pennsylvania, USA) were used in precipitation. Samples were run on 10% SDS-PAGE gel (Invitrogen, California, USA). The gel was fixed and incubated in EN3HANCE (Perkin Elmer, Massachusetts, USA) for 30 min and dried for X-ray film exposure.

**Supplement S1**

**Newly synthesized proteins, including Bcl-2, MMP-9 and IgG, detected in cultured cells with western blot**

The results showed that HPG was successfully incorporated into newly synthesized proteins (Fig 1B-b, lane 3), compared to negative control (Fig 1B-b, lane 1) and HPG incorporation into newly synthesized proteins was prevented by protein synthesis inhibitor, anisomycin (Fig 1B-b, lane 2). We extracted total proteins at various time points (0, 4, 24 and 72 hr) after incubating cultured cells with HPG for 4 hr and detected the biotin signals from newly synthesized total proteins after labeling reaction to test the specificity of the approach, the results showed that biotin signal strength from total proteins had changed responding to various time. Especially, biotin signal strength reached its peak after 4 hr after the change of medium supplemented with HPG into normal medium (Fig 1B-a, 4 hr). However, biotin signal strength of total proteins declined after 24 and 72 hr (Fig 1B-a, 24 and 72 hr). It meant that protein synthesis reached its peak after 4 hr and subsequent declined after 24 and 72 hr. Biotin signals of Bcl-2, MMP-9 and IgG pulled down by immunoprecipitation were detected with streptavidin-HRP by western blot. Our data showed that HPG was incorporated into newly synthesized Bcl-2 (26kD), MMP-9 (92kD) and IgG (25 and 50kD) (Fig 1B-c, lane 2), compared to control (Fig 1B-c, lane 1). To confirm that HPG was specifically incorporated into newly synthesized Bcl-2, MMP-9 and IgG proteins, non-radioactive metabolic labeling combined with immunoprecipitation and western blot was performed after siRNA silencing. Bcl-2 and MMP-9 proteins were both immunoprecipitated from labeled total protein with primary antibodies and protein G coupled with magnets, and IgG was directly immunoprecipitated with protein G coupled with magnets. Biotin signals of Bcl-2, MMP-9, and IgG were individually detected with western blot analysis and streptavidin-HRP. The results showed that the biotin signals of Bcl-2 protein band (26kD) and MMP-9 protein band (92kD) in the siRNA transfection group were declined from the control experiment in which a scrambed sequence instead of specific inhibitory sequences was employed (Fig 1B-c, d). The 50 kD band strength of IgG in the siRNA treated group was also decreased in comparison to the controls (Fig 1B, e). Radioactive pulse chase assay demonstrated that IgG was synthesized in TEV-1 cells, further confirming the validaty of this approach (Fig 1B, f). All the results demonstrated that HPG was specifically incorporated into newly synthesized Bcl-2, MMP-9, and IgG in cultured cells.

**Supplement S2**

**^35^S-methionine labeled IgG detected by autoradiography**

To further prove that IgG was actually synthesized in TEV-1 cells, radioactive labeling of pulse-chase assay was performed and IgG synthesis was confirmed in TEV-1 cells. ^35^S-methionine was incorporated into newly synthesized IgG (50kD and 25kD), which indicated a de novo synthesis of IgG in human choriocarcinoma cell line (Fig 1B, f, lane 1). Cycloheximide treatment further confirmed IgG synthesis since its inhibition on protein translational elongation significantly decresed radio-labeled IgG synthesis (50kD and 25kD) (Fig 1B, f, lane 2). No IgG synthesis was detected in human skin fibroblasts (Fig 1B, f, lane 3). Therefore, the results above demonstrated IgG synthesis mechanism existed in human choriocarcinoma cells and also confirmed that the non-radioactive metabolic method we provided was used to detect IgG synthesis.

**Supplement S3**

**IgG mRNA was detected in TEV-1 cells by RT-PCR**

To further prove IgG mRNA was transcribed in TEV-1 cells, we detected IgG mRNA expression by nested RT-PCR. The primers for IgG as follows: External 5’ -ACGGCGTGGAGGTGCATAATG-3’ (sense) and 5’-CGGGAGGCGTGGTCTTGT-

AGTT-3’ (antisense) and internal 5’-GACTGGCTGAATGGCAAGGAG-3’ (sense) and 5’-GGCGATGTCGCTGGGATAGAA-3’ (antisense). The size of PCR product for IgG was 201 bp [3]. The size of PCR product for GAPDH was 107 bp [4].The primers for GAPDH as follows: 5’-CCAGGTGGTCTCCTCTGACTTC-3’ (sense) and 5’-TCATACCAGGAAATGAGCTTGACA-3’ (antisense). The result demonstrated IgG mRNA was transcribed in TEV-1 cells (Supplement Figure S2).

**Supplement S4**

**Levels of Bcl-2, MMP-9, and IgG mRNA in cultured cells detected with real time RT-PCR after siRNA transfection**

Total cellular RNA was extracted after siRNA transfection. Bcl-2, MMP-9, and IgG mRNAs were detected with real time RT-PCR. The results showed that mRNA levels of the three target genes were significantly reduced following specific siRNA transfection. Compared to the scrambled siRNA negative controls, Bcl-2 mRNA was reduced by 66.6%, MMP-9 mRNA by 65.4% and IgG mRNA by 73.9%. The results demonstrated the effectiveness and specificity of siRNA oligos inhibition. This method was used to ensure the specificity of the reaction in the new technique. This control is only needed when establishing the technique but not required when practically applying the technique to detect the newly synthesized protein of interests.

**Supplement S5**

**Statistical analysis**

Statistical analysis of real time RT-PCR for Bcl-2, MMP-9 and IgG was performed with student’s *t*-test with two-tail in Excel software and the results were as follows: p=0.018 for Bcl-2, p=0.002 for MMP-9 and p=0.015 for IgG, all showed statistical significance.

We selected non-overlapping cells in the siRNA and scramble groups to further analyze the percentage of immunofluorescence co-localization for Alex555 and Alex488 with the Immaris software. The number of cells we measured was as follows: 14 cells from Bcl-2 siRNA slide and 14 cells from Bcl-2 scramble slide; 12 cells from MMP-9 siRNA slide and 11 cellls from MMP-9 scramble slide, and 12 cells from IgG siRNA slide and 12 cells from IgG scramble slide. Statistical analysis of the percentage of immunofluorescence co-localization of Bcl-2, MMP-9, and IgG were performed with student’s *t*-test, and the results were shown in table 2.

**Figure S1 and Figure legend**


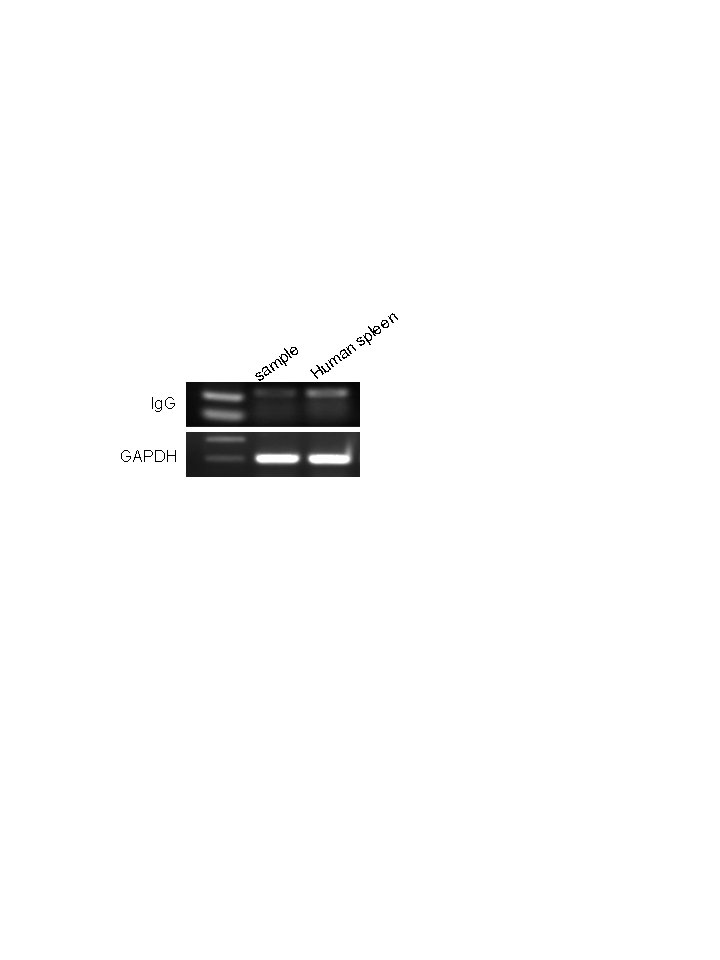


RT-PCR detection of IgG mRNA in TEV-1 cells by 1% gel electrophoresis. Sample was RNA extracted from TEV-1 cells. Human spleen was a positive control. GAPDH was an internal control.

**Reference**

[1] Hsu TL, Hanson SR, Kishikawa K, Wang SK, Sawa M, Wong CH: **Alkynyl sugar analogs for the labeling and visualization of glycoconjugates in cells.** *Proc Natl Acad Sci USA* 2007, **8:** 2614-2619.

[2] Yang C, Asthagiri AR, Iyer RR, Lu J, Xu DS, Ksendzovsky A, Brady RO, Zhuang Z, Lonser RR: **Missense mutations in the NF2 gene result in the quantitative loss of merlin protein and minimally affect protein intrinsic function.** *Proc Natl Acad Sci USA* 2011, **12:** 4980-4985.

[3] Hashimoto K, Kokubun S, Itoi E, Roach HI: **Improved quantification of DNA methylation using methylation-sensitive restriction enzymes and real-time PCR.** *Epigenetics* 2007, **2:** 86-91.

[4] Chen Z, Gu J: **Immunoglobulin G expression in carcinomas and cancer cell lines.** *FASEB J* 2007, **11:** 2931-2938.
